# Posterior Capsular Opacification in Preschool- and School-Age Patients after Pediatric Cataract Surgery without Posterior Capsulotomy

**DOI:** 10.4274/tjo.24650

**Published:** 2016-10-17

**Authors:** Muhammed Batur, Adem Gül, Erbil Seven, Ertuğrul Can, Tekin Yaşar

**Affiliations:** 1 Yüzüncü Yıl University Faculty of Medicine, Department of Ophthalmology, Van, Turkey; 2 Ondokuz Mayıs University Faculty of Medicine, Department of Ophthalmology, Samsun, Turkey

**Keywords:** Pediatric cataract, posterior capsular opacification, posterior capsulotomy

## Abstract

**Objectives:**

We aimed to evaluate the development of posterior capsular opacification (PCO) in preschool- and school-age children with cataract who underwent cataract surgery without posterior capsulotomy and anterior vitrectomy.

**Materials and Methods:**

The records of 30 eyes of 21 patients who underwent pediatric cataract surgery and intraocular lens (IOL) implantation were retrospectively reviewed. Patients’ age, PCO status and duration, need for neodymium-doped yttrium aluminium garnet (Nd:YAG) laser treatment based on coverage of visual axis, and follow-up period were recorded.

**Results:**

The mean age of the patients was 7.6±2.83 (4-12) years. Unilateral cataract surgery and IOL implantation were performed in 12 patients (57.14%) and bilateral cataract surgery and IOL implantation were performed in nine patients (42.86%). Average follow-up time was 17.7±22.67 (3-83) months. PCO developed in 21 eyes (70%) and covered the visual axis in 15 eyes (50%), which therefore required Nd:YAG laser posterior capsulotomy. The mean duration of postoperative PCO development was 8.91±18.7 months (1 week-71 months).

**Conclusion:**

We believe that with adequately experienced surgeons, performing both cataract surgery and posterior capsulotomy with anterior vitrectomy in the same session is appropriate for selected preschool- and school-age children with cataract.

## INTRODUCTION

Despite substantial improvements in cataract surgery techniques and intraocular lenses (IOLs), posterior capsular opacification (PCO) continues to be the most frequent postoperative complication of pediatric cataract surgery. Age is the main factor in PCO development, with the incidence increasing as age decreases. PCO development has been reported in up to 100% of infants after cataract surgery.^1^

Performing posterior capsulotomy and anterior vitrectomy in the same surgical session as cataract extraction is effective in preventing PCO obscuring the optical axis. However, in necessary cases and with cooperative patients, especially school-aged children whose posterior capsule is intact, neodymium-doped yttrium aluminium garnet (Nd:YAG) laser capsulotomy is another treatment option for PCO. In patients not suitable for laser therapy, anterior vitrectomy and posterior capsulotomy can be performed via the pars plana approach.^2,3,4^

Nd:YAG laser therapy has certain limitations, including transient intraocular pressure (IOP) elevation, high cost, limited access to the instruments, noncompliance with laser therapy in young children, need for general anesthesia and the high incidence of IOL damage. Furthermore, even in the absence of the posterior capsule, residual lens fibers may migrate to the intact vitreous surface and form secondary opaque membranes.^5^

Performing posterior capsulotomy and anterior vitrectomy in the same surgical session as cataract extraction also has some disadvantages. These include longer surgery time, requirement of more experience and skill on the part of the surgeon, vitreous loss, IOL dislocation, and higher rates of cystoid macular edema and retinal detachment.^2,3,4^ As a result, surgeons face several questions. At what age should patients’ posterior capsule be opened and when should it be left intact? At what age should posterior capsulotomy and anterior vitrectomy be performed together?

In this study we aimed to evaluate PCO status and treatment required due to PCO obscuring the optical axis in patients 4-12 years of age who underwent pediatric cataract surgery without posterior capsulotomy or anterior vitrectomy.

## MATERIALS AND METHODS

The charts of pediatric patients who underwent cataract surgery and IOL implantation at Yüzüncü Yıl University Faculty of Medicine, Department of Ophthalmology (between 2006-2013, n=18) and Ondokuz Mayıs University Faculty of Medicine, Department of Ophthalmology (between 2012-2013, n=12) were analyzed retrospectively.

Patients whose operations did not include posterior capsulotomy and anterior vitrectomy, who were between 4 and 12 years old and had non-traumatic cataract were included in the study. Thirty eyes of 21 patients were included.

Retinoscopy, biomicroscopy and dilated fundus examinations were done preoperatively in some patients based on extent of cataract and postoperatively in all patients. Pre- and postoperative best corrected visual acuity (BCVA) of compliant patients was recorded (Snellen). The diopter, brand and implantation location was recorded for implanted IOLs. Cataract type was evaluated.

Surgical technique: The anterior chamber was accessed by clear corneal incision. After staining the anterior capsule with trypan blue, continuous anterior capsulotomy of 5-5.5 mm diameter was performed under viscoelastic. Two side ports were created. The lens material was aspirated using bimanual irrigation/aspiration handpieces. The IOL was implanted in the sulcus in 2 patients (6.67%) and in the capsule in the remaining patients. The corneal incision was widened in some patients to accommodate the implantation of polymethyl methacrylate (PMMA) IOLs. The viscoelastic in the anterior chamber and under the IOL was then aspirated using irrigation/aspiration and the corneal incision was sutured with 10-0 nylon. The procedure was concluded with diluted cefuroxime injection into the anterior chamber and subconjunctival dexamethasone injection.

After the procedure, patients used 1% prednisolone drops 8 times a day, ofloxacin drops 8 times a day, 1% cyclopentolate drops twice a day and systemic 1 mg/kg methylprednisolone for 3 days. Topical drops were tapered and discontinued within 1 month. Postoperative follow-up examinations were done at 1 day, 1 week, 1 month, and 3 months after the procedure, and thereafter at fixed intervals determined based on the patient’s condition. PCO development, PCO duration, need for Nd:YAG laser therapy due to optical axis obscuration and follow-up time were recorded at follow-up visits.

## RESULTS

Mean age of the patients was 7.6±2.83 (4-12) years. Twelve (57.14%) of the patients had unilateral cataract surgery (6 right, 6 left eyes) and 9 patients (42.86%) had bilateral cataract surgery. Cataract types are given in Table 1.

Mean power of implanted IOLs was 24.67±6.11 (8-31) diopters.

PCO developed in 21 eyes (70%) and obscured the optic axis to an extent that required capsulotomy by Nd:YAG laser in 15 eyes (50%). IOL types and PCO status are shown in Table 2.

Mean follow-up time was 17.7±22.67 (3-83) months. Postoperative time to PCO development was 8.91±18.7 months (1 week-71 months).

Preoperative visual acuity could not be determined in 6 patients (20%). Pre- and postoperative BCVA values are shown in Table 3.

## DISCUSSION

Pediatric cataract is one of the leading causes of preventable blindness in children. As visual development is ongoing in children, pediatric cataracts do not only impact vision but also impair normal visual development, leading to amblyopia, strabismus or nystagmus.^6^ Children are particularly susceptible to amblyopia in the first 2-3 years of life. There is a better chance of successfully treating amblyopia that develops after the age of 4. The critical period is considered to continue until 6-12 years of age, though this period may vary for different visual functions.^7^

There are several factors that affect the incidence of PCO development after pediatric cataract surgery. These factors include surgical age, accompanying ocular pathologies, extent of cortex clearance, surgical management of the posterior capsule and anterior vitreous, IOL parameters (design, material and location) and surgical trauma.^5^

Ensuring a clear visual axis after pediatric cataract surgery is crucial for good visual acuity results. In young children, the inflammatory response is very intense and the visual axis may be obscured by fibrous membranes proliferating on the intact anterior vitreous surface.^5^ Opacification obscuring the visual axis is a common postoperative complication of pediatric cataract surgery. The rate of PCO development can reach 100% in children younger than 4 years old when the posterior capsule is intact.^1^

Various surgical procedures are utilized to prevent PCO. Opacity in the optic axis has been reported in up to 60% of patients who had primary posterior capsulotomy without anterior vitrectomy.^8,9^ Some researchers have reported the lens epithelial cells and their remnants formed a basis for proliferation on the anterior hyaloid surface.^10^ Opacity in the optic axis has been reported at rates less than 20% when anterior vitrectomy and posterior capsulotomy are performed together with cataract surgery.^4,11,12^ Despite anterior vitrectomy, this opacity arose due to insufficient posterior capsule opening and anterior vitrectomy.^11^

Another technique used to prevent PCO is optic capture, in which the IOL haptics are positioned inside the capsule after the primary posterior capsulotomy (with or without anterior vitrectomy), thus fixing the optics posterior to the capsule. This prevents the proliferation of lens epithelial cells on the anterior vitreous surface. However, lens epithelial cells may migrate from the IOL haptic-optic junction and proliferate on the posterior of the capsule and the IOL surface. Therefore, this technique may also fail to completely prevent secondary membrane development.^5^

Gimbel et al.^13^ expressed concerns that performing vitrectomy in the eyes of children negatively impacts ocular development. However, anterior vitrectomy should be performed with primary posterior capsulotomy in infants and young children due to the risk of amblyopia and high PCO incidence. For older children, Nd:YAG laser capsulotomy can be considered.^14^

There is no consensus on the age range within which posterior capsulotomy should be performed in the same surgical session as cataract extraction. Luo et al.^15^ recommended up to the age of 5 years, Jensen et al.^2^ recommended up to 6 years old, Vasavada et al.^5^ up to 6 or 7 years old, and Guo et al.^16^ up to 10 years old. Astle et al.17 found that the PCO rate decreased with age from 70.8% in children less than 1 year old to 6.1% in children older than 7 years old. In practice, we generally perform posterior capsulotomy and anterior vitrectomy in our cataract surgeries for children younger than 6 years of age. However, in PCO cases we believe will cooperate with Nd:YAG laser treatment, we prefer performing cataract surgery without posterior capsulotomy.

In a study using acrylic IOLs (AcrySof) in children aged 2-16 years, PCO developed in 83.8% (27.7% requiring treatment) of patients that did not undergo posterior capsulotomy, 37.5% (7.5% requiring treatment) of patients that had posterior capsulotomy without vitrectomy, and 6.7% (treatment was not necessary) of patients that underwent both vitrectomy and posterior capsulotomy.^12^ The authors also reported a significantly higher rate of PCO development in children 8 years old or younger compared with children over 8 years old (p=0.01).^12^ In another study, 9 of 21 pediatric patients who underwent cataract surgery without posterior capsulotomy developed PCO and 7 of those patients required Nd:YAG laser therapy.^18^

Luo et al.^15^ divided congenital cataract patients aged 2-5 years into 2 groups and determined PCO rate as 11.8% in the group that had posterior capsulotomy with vitrectomy versus 76.9% in the group that had cataract extraction. The rate of Nd:YAG laser capsulotomy was 2.9% and 57.7% in the groups, respectively.

Jafarinasap et al.^19^ reported that of 9 patients aged 10-15 years who underwent lensectomy and posterior chamber IOL (Alcon AcrySof MA60 AC) implantation, 3 patients developed PCO but none required treatment, while none of the 8 patients that had anterior vitrectomy and posterior capsulotomy during lensectomy surgery developed PCO.

Aasuri et al.^20^ implanted an acrylic IOL in one eye and a PMMA IOL in the fellow eye of bilateral cataract patients aged 5 years and older. Clinically significant PCO rates were reported in both the acrylic IOL group (21%) and the PMMA group (75%).

We consider the PCO rate determined in our study consistent with data from the published studies cited above. A limitation of our study was the lack of PCO development data from a group of patients that underwent posterior capsulotomy (and anterior vitrectomy) during their cataract surgery.

Although Nd:YAG laser capsulotomy is used to treat PCO safely and effectively, it is not without complications. There have been reports of serious complications such as retinal edema and retinal detachment as well as other complications like IOP elevation, vitreous prolapse, corneal damage, vitritis, pupil block, hyphema, and IOL damage and dislocation.^21^

### Study Limitations

Limitations of Nd:YAG laser therapy include high cost, limited access to the instruments, noncompliance with laser therapy in young children, and the need for general anesthesia. Furthermore, even without the posterior capsule, residual lens fibers may migrate to the intact vitreous surface and form secondary opaque membranes. Hutcheson et al.^4^ reported that visual axis obscuring opacity recurred at a rate of 57% after Nd:YAG laser capsulotomy and a third laser treatment was required in 17% of cases.

The inability to prevent PCO is the greatest difficulty faced by pediatric ophthalmologists worldwide. The development (for pediatric eyes) of medical antagonists against factors leading to PCO or capsule washing substances that reduce the speed and severity of lens epithelial cell proliferation would solve this problem.^14^

## CONCLUSION

Young children have a high rate of PCO development, making cataract extraction with posterior capsulotomy (and anterior vitrectomy) appropriate for this age group. We believe that with adequately experienced surgeons, posterior capsulotomy and anterior vitrectomy should be performed in the same surgical session as cataract extraction in selected preschool- and school-age children.

### Ethics

Ethics Committee Approval: The study were approved by the Van Yüzüncü Yıl University of Local Ethics Committee (protocol number: 10, date: 10.03.2015), Informed Consent: Not needed.

Peer-review: Externally peer-reviewed.

## Figures and Tables

**Table 1:**
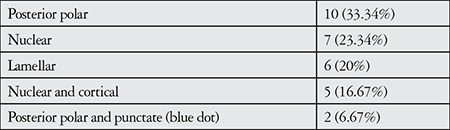
Distribution of cataract types [n, (%)]

**Table 2:**
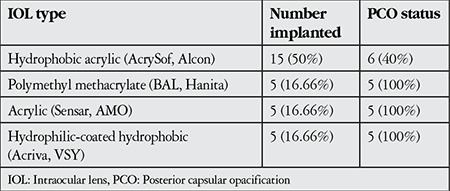
Types of intraocular lens implanted and posterior capsular opacification status [n (%)]

**Table 3:**
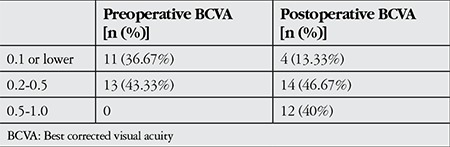
Pre- and postoperative best corrected visual acuity levels (Snellen)

## References

[1] Spierer A, Desatnik H, Blumenthal M (1999). Refractive status in children after long-term follow up of cataract surgery with intraocular lens implantation. J Pediatr Ophthalmol Strabismus.

[2] Jensen AA, Basti S, Greenwald MJ, Mets MB (2002). When may the posterior capsule be preserved in pediatric intraocular lens surgery?. Ophthalmology.

[3] Guo S, Wagner RS, Caputo A (2004). Management of the anterior and posterior lens capsules and vitreous in pediatric cataract surgery. J Pediatr Ophthalmol Strabismus.

[4] Hutcheson KA, Drack AV, Ellish NJ, Lambert SR (1999). Anterior hyaloid face opacification after pediatric Nd:YAG laser capsulotomy. J AAPOS.

[5] Vasavada AR, Praveen MR, Tassignon MJ, Shah SK, Vasavada VA, Vasavada VA, Van Looveren J, De Veuster I, Trivedi RH (2011). Posterior capsule management in congenital cataract surgery. J Cataract Refract Surg.

[6] Uzun A, Atilla H (2013). Pediatrik katarakt kedeniyle opere edilen çocuklarda hayat kalitesi. Turk J Ophthalmol.

[7] Koçak G, Duranoğlu Y (2014). Ambliyopi ve tedavisi. Turk J Ophthalmol.

[8] Morgan KS, Karcioglu ZA (1987). Secondary cataracts in infants after lensectomies. J Pediatr Ophthalmol Strabismus.

[9] Nishi O (1988). Fibrinous membrane formation on the posterior chamber lens during the early postoperative period. J Cataract Refract Surg.

[10] Jones NP, McLeod D, Boulton ME (1995). Massive proliferation of lens epithelial remnants after Nd-YAG laser capsulotomy. Br J Ophthalmol.

[11] Alexandrakis G, Peterseim MM, Wilson ME (2002). Clinical outcomes of pars plana capsulotomy with anterior vitrectomy in pediatric cataract surgery. J AAPOS.

[12] Vasavada AR, Trivedi RH, Nath VC (2004). Visual axis opacification after AcrySof intraocular lens implantation in children. J Cataract Refract Surg.

[13] Gimbel HV, Ferensowicz M, Raanan M, DeLuca M (1993). Implantation in children. J Pediatr Ophthalmol Strabismus.

[14] Medsinge A, Nischal KK (2015). Pediatric cataract: challenges and future directions. Clin Ophthalmol.

[15] Luo Y, Lu Y, Lu G, Wang M (2008). Primary posterior capsulorhexis with anterior vitrectomy in preventing posterior capsule opacification in pediatric cataract microsurgery. Microsurgery.

[16] Guo S, Wagner RS, Caputo A (2004). Management of the anterior and posterior lens capsules and vitreous in pediatric cataract surgery. J Pediatr Ophthalmol Strabismus.

[17] Astle WF, Alewenah O, Ingram AD, Paszuk A (2009). Surgical outcomes of primary foldable intraocular lens implantation in children: understanding posterior opacification and the absence of glaucoma. J Cataract Refract Surg.

[18] Er H, Doganay S, Evereklioglu C, Erten A, Cumurcu T, Bayramlar H (2000). Retrospective comparison of surgical techniques to prevent secondary opacification in pediatric cataracts. J Pediatr Ophthalmol Strabismus.

[19] Jafarinasab MR, Rabbanikhah Z, Karimian F, Javadi MA (2008). Lensectomy and PCIOL Implantation with versus without posterior capsulotomy and anterior vitrectomy for pediatric cataracts. J Ophthalmic Vis Res.

[20] Aasuri MK, Fernandes M, Pathan PP (2006). Comparison of acrylic and polymethyl methacrylate lenses in a pediatric population. Indian J Ophthalmol.

[21] Karahan E, Er D, Kaynak S (2014). An Overview of Nd:YAG Laser Capsulotomy. Med Hypothesis Discov Innov Ophthalmol.

